# The effect of the muscle environment on the regenerative capacity of human skeletal muscle stem cells

**DOI:** 10.1186/s13395-015-0036-8

**Published:** 2015-04-28

**Authors:** Jinhong Meng, Maximilien Bencze, Rowan Asfahani, Francesco Muntoni, Jennifer E Morgan

**Affiliations:** The Dubowitz Neuromuscular Centre, Molecular Neurosciences Section, Developmental Neurosciences Programme, UCL Institute of Child Health, 30 Guilford Street, London, WC1N 1EH UK

**Keywords:** Human skeletal muscle stem cells, Transplantation, Animal model, Immunodeficiency, Rag2-/γ chain-/C5- mice, *mdx* nude mice, Stem cell therapy, Satellite cells

## Abstract

**Background:**

Muscle stem cell transplantation is a possible treatment for muscular dystrophy. In addition to the intrinsic properties of the stem cells, the local and systemic environment plays an important role in determining the fate of the grafted cells. We therefore investigated the effect of modulating the host muscle environment in different ways (irradiation or cryoinjury or a combination of irradiation and cryoinjury) in two immunodeficient mouse strains (*mdx* nude and recombinase-activating gene (Rag)2-/γ chain-/C5-) on the regenerative capacity of two types of human skeletal muscle-derived stem cell (pericytes and CD133+ cells).

**Methods:**

Human skeletal muscle-derived pericytes or CD133+ cells were transplanted into muscles of either *mdx* nude or recombinase-activating gene (Rag)2-/γ chain-/C5- host mice. Host muscles were modulated prior to donor cell transplantation by either irradiation, or cryoinjury, or a combination of irradiation and cryoinjury. Muscles were analysed four weeks after transplantation, by staining transverse cryostat sections of grafted muscles with antibodies to human lamin A/C, human spectrin, laminin and Pax 7. The number of nuclei and muscle fibres of donor origin and the number of satellite cells of both host and donor origin were quantified.

**Results:**

Within both host strains transplanted intra-muscularly with both donor cell types, there were significantly more nuclei and muscle fibres of donor origin in host muscles that had been modulated by cryoinjury, or irradiation+cryoinjury, than by irradiation alone. Irradiation has no additive effects in further enhancing the transplantation efficiency than cryodamage. Donor pericytes did not give rise to satellite cells. However, using CD133+ cells as donor cells, there were significantly more nuclei, muscle fibres, as well as satellite cells of donor origin in Rag2-/γ chain-/C5- mice than *mdx* nude mice, when the muscles were injured by either cryodamage or irradiation+cryodamage.

**Conclusions:**

Rag2-/γ chain-/C5- mice are a better recipient mouse strain than *mdx* nude mice for human muscle stem cell transplantation. Cryodamage of host muscle is the most effective method to enhance the transplantation efficiency of human skeletal muscle stem cells. This study highlights the importance of modulating the muscle environment in preclinical studies to optimise the efficacy of transplanted stem cells.

**Electronic supplementary material:**

The online version of this article (doi:10.1186/s13395-015-0036-8) contains supplementary material, which is available to authorized users.

## Background

Muscular dystrophies are a group of inherited diseases characterised by muscle weakness and wasting. A common and severe form of muscular dystrophy is Duchenne muscular dystrophy (DMD), caused by mutations in the dystrophin gene. Typical pathological changes within the muscles of a DMD patient include progressive degeneration and regeneration of muscle fibres, accompanied by the exhaustion of muscle-resident stem cells such as satellite cells, leading to a net loss of muscle fibres that are eventually replaced by fibro-fatty tissue [1]. Transplantation of stem cells has been suggested as a promising way to treat DMD, as donor cells would repair and regenerate muscle fibres; stem cells derived from normal donors would also restore dystrophin expression within these regenerated muscle fibres. If the donor cells also formed functional satellite cells to replenish the muscle stem cell pool, this should provide a long-term source of fibres in DMD patients.

However, stem cells need to be extensively tested in laboratory animal models to elucidate their suitability for clinical application, and it is important that an appropriate animal model is used. Different types of dystrophin-deficient [2-6] or non-dystrophic host mice [7-13] have been used for this purpose. For donor stem cells of human origin, this represents xenografting, which requires the host mouse to be profoundly immunodeficient. To augment engraftment of intra-muscularly transplanted human as well as mouse muscle stem cells, the host muscle needs to be modulated prior to cell transplantation. Although the needle used to deliver donor cells intra-muscularly does cause local injury, this may not be sufficient to promote donor cell engraftment. For example, either freshly isolated mouse satellite cells or a single myofibre bearing satellite cells give rise to little, if any, muscle of donor origin after their transplantation into non-injured host *mdx* nude mouse muscles [14,15]. Although mouse myoblasts do give rise to regenerated muscle fibres in non-injured *mdx* nude or recombinase-activating gene (Rag)2-/γ chain-/C5- host muscles, they form significantly less muscle than when grafted into muscles in mice of both strains that had been irradiated with 18 Gy 3 days before grafting [16]. Human myoblasts also gave rise to less muscle of donor origin when transplanted into non-injured compared to cryoinjured host muscles [6,7].

In a preliminary study, we injected human skeletal muscle-derived CD133+ cells or pericytes into non-injured host *mdx* nude (*n* = 2) or Rag2-/γ chain-/C5- (*n* = 2) mouse muscles, and no cells or muscle fibres of donor origin were detected in these grafted muscles (data not shown). Thus, we considered that the modulation to the host muscle induced by needle injury and cell injection is not sufficient to elicit effective engraftment of human muscle stem cells. Indeed, different muscle pre-treatment regimes have been used by different groups, including irradiation [7,14-17], cryoinjury [11,18,19] or injection of myotoxin [20,21]. The precise mechanism underlying the effect of these pre-treatments is not completely clear. In one study, the effect of pre-injury of host muscle with cardiotoxin or cyoinjury on human myoblast engraftment in host muscles was investigated [12], but no systematic comparison has been performed by using different modalities to modulate the engraftment efficiency of human stem cells within host muscles.

In addition, the effect of the host mouse strain on donor stem cell engraftment is not known. The Rag2-/γ chain-/C5- mouse is a triple knockout immunodeficient mouse that has been used for human myoblast transplantation studies [7,12,22,23]. The lack of recombinase-activating gene Rag2 results in total inability to initiate V(D)J rearrangement, leading to a severe combined immune deficient (SCID) phenotype [24]. Deletion of the common cytokine receptor γ chain gene results in reduced numbers of peripheral T and B lymphocytes and absence of natural killer (NK) cell activity [25,26]. A further deletion of the C5 component of the complement cascade leads to a defect in innate immunity [12]. The combination of knocking out these three molecules makes this mouse an ideal animal model for xenografts, including human muscle stem cell transplantation [7,12,22,23], but the mouse is not dystrophic.

An alternative to test the efficacy of donor stem cells to treat a muscular dystrophy is a mouse model of a human muscular dystrophy, which is also immunodeficient. The *mdx* mouse lacks dystrophin in skeletal muscles body-wide and is a much-used model of DMD [27,28]. However, it has rare, naturally occurring revertant fibres [29,30] that have to be taken into account when assessing donor stem cell-mediated restoration of dystrophin. The *mdx* mouse has been put on immunodeficient backgrounds including nude [16,31] and SCID [3,4]. More recently, two novel immunodeficient dystrophin-deficient mouse models were developed as hosts for cell transplantation that had either fewer revertant fibres than *mdx* [2] or a complete absence of dystrophin [6].

The *mdx* nude mouse has been used extensively as recipient animal model to analyse the regenerative capacity of muscle stem cells of mouse [14,15,27,31-33] and human origin [5]. However, the nude mouse lacks the majority of the T-cells due to the deletion of Foxn1 (winged-helix/forkhead transcription factor) gene [34] but maintains normal B cell function and high NK cell activity, thus is less immunodeficient than the Rag2-/γ chain-/C5- mouse.

In this study, we aimed to determine whether and to what extent the modulation of host muscle environment would affect the outcomes of stem cell transplantation. To do this, we used two types of stem cell derived from human skeletal muscle - human skeletal muscle-derived stem cells, or pericytes [5], and CD133+ cells [10], and compared their transplantation efficiency in Rag2-/γ chain-/C5- and *mdx* nude mouse muscles that had been modified by either irradiation, or cryoinjury, or a combination of both. Our results show that the ability of the grafted human stem cells to participate in muscle regeneration and to reconstitute the satellite cell pool is dependent on both the recipient mouse strain and the way in which the host muscle is modulated prior to cell transplantation.

## Methods

### Ethics

Human cells were obtained from the MRC Centre for Neuromuscular Diseases Biobank. Tissue sampling was approved by the NHS National Research Ethics Service, Hammersmith and Queen Charlotte’s and Chelsea Research Ethics Committee: setting up of a rare diseases biological samples bank (biobank) for research to facilitate pharmacological, gene and cell therapy trials in neuromuscular disorders (REC reference number 06/Q0406/33) and the use of cells as a model system to study pathogenesis and therapeutic strategies for Neuromuscular Disorders (REC reference 13/LO/1826), in compliance with national guidelines regarding the use of biopsy tissue for research. All patients or their legal guardians gave written informed consent for the collection and use of their cells.

Animal experiments were approved by University College London’s Animal Welfare Ethical Review Body. Mice were bred and experimental procedures were carried out in the Biological Services Unit, University College London Institute of Child Health, in accordance with the Animals (Scientific Procedures) Act 1986. Experiments were performed under Home Office licence 70/7086.

### Human skeletal muscle-derived stem cells

Human pericytes were isolated from the extensor digitorum brevis muscle of an 11-year-old DMD patient, as described previously [5]. Human skeletal muscle-derived CD133+ cells were isolated from the para-spinal muscle of a 15-year-old boy with adolescent idiopathic scoliosis, using a previously described protocol [3,10].

Both types of cell were maintained *in vitro* in Megacell DMEM (Sigma-Aldrich, Dorset, UK) containing 10% fetal bovine serum (FBS) (Invitrogen, Paisley, UK). Pericytes were transplanted at mean population doubling (mpd) 13.58 to 18.90 and CD133+ cells at mpd 6.8 to 7.91(Additional file 1: Table S1).

### Mouse strains and muscle injury models

Host mice aged 4 to 8 weeks were used for this study. Two immunodeficient mouse strains, Rag2-/γ chain-/C5- [16,23] and *mdx* nude [16,31] mice, were used as hosts.

Host tibialis anterior (TA) muscles were modulated in three different ways prior to the injection of donor cells.

Irradiation: 3 days before intramuscular transplantation of donor cells, mice were anaesthetised with hypnorm and hypnovel and 18 Gy radiation, at a dose rate of 0.72 Gy/min, was administered to the hind limbs [35].

Cryodamage: immediately prior to donor cell transplantation, host mice were anaesthetised with isoflurane and TA muscles cryoinjured, as described previously [7].

Irradiation+cryodamage: hind limbs were irradiated with 18 Gy as described above and then 3 days later, TA muscles were cryoinjured immediately prior to cell transplantation.

### Donor cell transplantation

In 5 μl of medium, 5 × 10^5^ human muscle stem cells were injected into each host TA muscle, using a 5-μl Hamilton syringe.

For human muscle stem cell transplantation, four sets of experiments were performed using both types of muscle stem cell (Additional file 1: Table S1).

### Analysis of grafted muscles

Four weeks after transplantation, grafted muscles were removed for analysis. Ten-micrometer transverse sections were cut throughout the muscle with a Leica cryostat 1850, mounted onto Polysine adhesion slides (VWR, Leicestershire, UK) and stored at −80°C.

Immunostaining with antibodies to human lamin A/C (Vector Laboratories, Peterborough, UK) and human spectrin (Vector Laboratories, Peterborough, UK) was performed as described previously [5,7,9]. For staining of sections of *mdx* nude muscles, mouse Ig blocking reagent (Vector Laboratories, Peterborough, UK) was added in the blocking solution according to the manufacturer’s instructions. Muscle sections were incubated with primary antibodies for 1 h, followed by corresponding secondary antibodies for another hour, then were mounted with mounting medium (DAKO, Ely, UK) containing 10 μg/ml 4′,6-diamidino-2-phenylindole (DAPI). Images were captured using Metamorph software using a Leica microscope.

For multi-channel immunostaining of human lamin A/C, human spectrin, Pax7 (1:100, DSHB, Iowa City, Iowa) and pan-laminin (1:2,000, Sigma-Aldrich, Dorset, UK), muscle sections were fixed with 4% paraformaldehyde (PFA) for 15 min at room temperature. The sections were then blocked as above and incubated with a combination of the primary antibodies for 1 h at room temperature, followed by a combination of Alexa-488 conjugated goat anti-mouse IgG1, Alexa-594 conjugated goat anti-mouse IgG2b and Alexa-647 conjugated goat anti-rabbit IgG (H + L) for 1 h (1:500 each, Invitrogen, Paisley, UK). Sections were then mounted and images were captured with a Zeiss LSM 710 confocal microscope (Carl Zeiss AG, Oberkochen, Germany).

### Quantification and statistical analysis

To determine donor cell number and their contribution to regenerated muscle fibres, the number of human lamin A/C+ nuclei and the number of human spectrin+fibres which contain at least one human lamin A/C+ nucleus [36] were counted in the section that contained the most fibres of donor origin in each transplanted muscle and compared with one-way ANOVA or Mann-Whitney test using PRISM 5 software.

The total number of satellite cells or myoblasts, and the number of these cells that was of donor or host origin, within muscles that had been grafted with CD133+ cells were determined. The number of Pax7+ cells (total), Pax7+/human lamin A/C+ cells (donor) and Pax7+/human lamin A/C- cells (host) was counted and normalized to one hundred myofibres (delineated by laminin immunostaining) in a representative section of each muscle and compared between the two mouse strains with Mann-Whitney test, using PRISM 5 software.

To identify *bona fide* satellite cells (defined by their position under the basal lamina of muscle fibres) formed by transplanted CD133+ cells, the number of Pax7+/human lamin A/C+ cells which were located underneath the basal lamina (delineated by pan-laminin staining) were counted and normalized to one hundred myofibres in a representative section of each muscle and compared between the two mouse strains with Mann-Whitney test using PRISM 5 software.

## Results

### The number of nuclei and muscle fibres of donor origin is affected by modulation of the host muscle prior to engraftment

To investigate the effects of host muscle injury on donor cell transplantation efficiency, we injected either human pericytes or CD133+ cells into either *mdx* nude or Rag2-/γ chain-/C5- host mouse muscles that had received either irradiation or cryodamage or irradiation+cryodamage, prior to donor cell transplantation.

### *mdx* nude hosts

When human pericytes was used as donor cells, there were 51.83 ± 20.01, 84.17 ± 13.97 and 110.4 ± 56.64 human lamin A/C+ cells in irradiated, cryodamaged and irradiated+cryodamaged muscles, with no significant difference among the three injury groups (*P* = 0.465, one-way ANOVA). However, there were significant differences in the number of human spectrin+fibres that contained at least one human lamin A/C+ nucleus (S+L fibres), which is a stringent criterion to identify muscle fibres of donor origin, among the three injury groups (*P* = 0.0214, one-way ANOVA). There were significantly more donor muscle fibres in cryodamaged (27.83 ± 6.421, *P* = 0.0060) or irradiated+cryodamaged (18.20 ± 7.933, *P* = 0.0267) than irradiated (3.00 ± 1.506) muscles.

When CD133+ cells were the donor cells, there were significant differences in the number of human lamin A/C+ nuclei among irradiated (32.50 ± 16.66), cryodamaged (248.0 ± 35.39) and irradiated+cryodamaged (195.2 ± 48.64) muscles (*P* = 0.0012, one-way ANOVA). There were significantly more donor cells in cryodamaged (*P* = 0.0050) or irradiated+cryodamaged (*P* = 0.0222) than irradiated muscles. Similarly, there were significant differences in the number of S+L fibres among the three injury groups (*P* = 0.0008, one-way ANOVA). There were significantly more donor fibres in cryodamaged (107.0 ± 14.14, *P* = 0.0050) or irradiated+cryodamaged (113.8 ± 25.39, *P* = 0.0102) than irradiated (14.50 ± 6.980) muscles.

**Figure 1:**
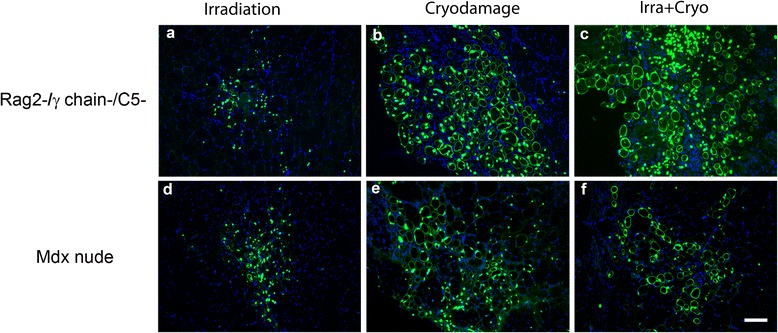
Representative images of transverse cryosections of regenerated muscles that had been grafted with CD133+ cells. Sections were stained with human lamin A/C and human spectrin antibodies (both green), and nuclei were counterstained with DAPI (blue). **(a), (b)** and **(c)** are sections from Rag2-/γ chain-/C5- mice, and **(d)**, **(e)** and **(f)** are sections from *mdx* nude mice. (a) and (d) were modulated prior to cell transplantation with irradiation only, (b) and (e) were modulated by cryodamage only, (c) and (f) were modulated with irradiation+cryodamage. Scale bar = 50 μm. Rag, recombinase-activating gene.

**Figure 2:**
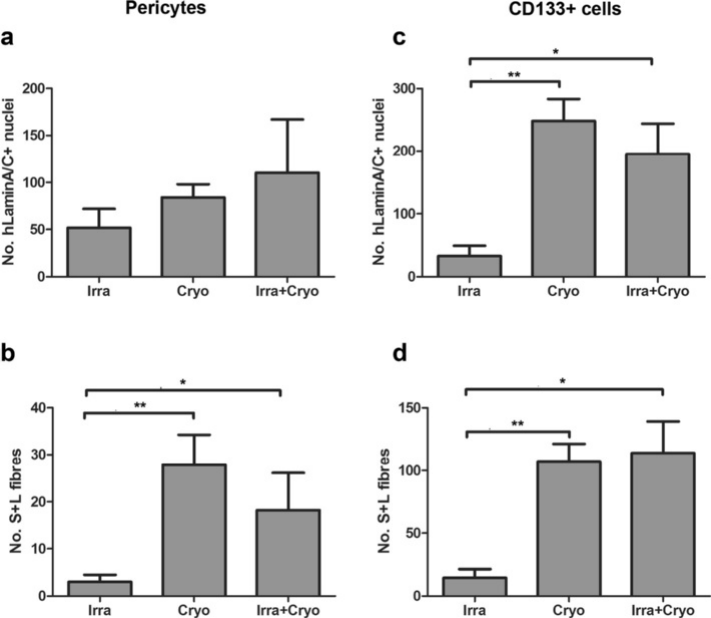
Donor cell/muscle fibre formation in *mdx* nude mice is affected by the way in which the host muscle is modulated prior to engraftment. Quantification and comparison of the number of human lamin A/C+ nuclei and fibres containing at least one human lamin A/C+ nucleus and expressing human spectrin (S+L fibres) derived from donor pericytes **(a, b)** or CD133+ cells **(c, d)** in muscles injured in different ways. *P < 0.05, **P < 0.01.

There were no significant differences in either the number of cells, or fibres, derived from either donor stem cell types between cryodamaged and irradiated+cryodamaged muscles (Table 1, Figures 1 and 2).

**Table 1:** **The transplantation efficiency of human muscle stem cells is affected by the type of injury**

**Donor cells**	**Donor contribution**	**Injury model**			***P*** **value (one-way ANOVA)**
**Host mouse**		**Number of donor nuclei or fibres (mean ± SEM (** ***n*** **))**			
		**Irradiation**	**Cryodamage**	**Irra+Cryo**	
Pericytes *mdx* nude	Human lamin A/C+ nuclei	51.83 ± 20.01 (6)	84.17 ± 13.97 (6)	110.4 ± 56.64 (5)	0.465
	S+L fibres	3.000 ± 1.506 (6)	27.83 ± 6.421 (6)	18.20 ± 7.933 (5)	0.0214*
CD133+ *mdx* nude	Human lamin A/C+ nuclei	32.50 ± 16.66 (6)	248.0 ± 35.39 (6)	195.2 ± 48.64(5)	0.0012**
	S+L fibres	14.50 ± 6.980 (6)	107.0 ± 14.14 (6)	113.8 ± 25.39 (5)	0.0008***
Pericytes Rag2-/γ chain-/C5-	Human lamin A/C+ nuclei	79.55 ± 31.13(11)	270.9 ± 76.12 (8)	270.1 ± 69.51 (11)	0.0424*
	S+L fibres	15.64 ± 6.33 (11)	132.6 ± 35.37 (8)	69.82 ± 21.01 (11)	0.0036**
CD133+ Rag2-/γ chain-/C5-	Human lamin A/C+ nuclei	98.83 ± 44.22 (6)	623.2 ± 117.3 (6)	569.3 ± 104.8 (6)	0.0023**
	S+L fibres	18.00 ± 6.583 (6)	191.3 ± 37.04 (6)	146.7 ± 18.38 (6)	0.0004***

*P < 0.05;**P < 0.01; ***P < 0.001.

### Rag2-/γ chain-/C5- hosts

Within muscles that had been grafted with pericytes, there were significant differences (*P* = 0.0424, one-way ANOVA) in the number of human lamin A/C+ cells among the three injury groups. There were significantly more human lamin A/C+ cells in cryodamaged (270.9 ± 76.12, *P* = 0.0286) or irradiated+cryodamaged (270.1 ± 69.51, *P* = 0.0416) than irradiated (79.55 ± 31.13) muscles. There were also significant differences (*P* = 0.0036, one-way ANOVA) in the number of S+L fibres among the three injury groups. There were significantly more donor fibres in cryodamaged (132.6 ± 35.37, *P* = 0.0032) or irradiated+cryodamaged (69.82 ± 21.01, *P* = 0.0207) than irradiated (15.64 ± 6.328) muscles.

Similarly, when CD133+ cells were transplanted, there were significant differences in the number of human lamin A/C+ nuclei (*P* = 0.0023, one-way ANOVA) and S+L fibres (*P* = 0.0004) among the three injury groups. There were significantly more human lamin A/C+ cells in cryodamaged (623.2 ± 117.3, *P* = 0.0043) or irradiated+cryodamaged (569.3 ± 104.8, *P* = 0.0043) than irradiated (51.83 ± 20.01) and there were also significantly more S+L fibres in cryodamaged (191.3 ± 37.04, *P* = 0.0050) or irradiated+cryodamaged (146.7 ± 18.38, *P* = 0.0050) than in irradiated (18.00 ± 6.583) muscles.

**Figure 3:**
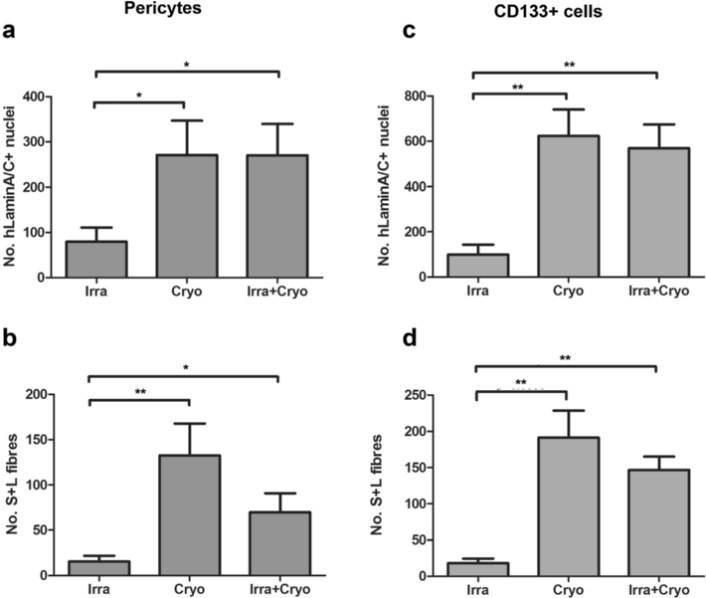
Donor cell/muscle fibre formation in Rag2-/γ chain-/C5- mice is affected by the way in which the host muscle is modulated prior to engraftment. Quantification and comparison of the number of human lamin A/C+ nuclei and S+L fibres derived from donor pericytes **(a, b)** or CD133+ cells **(c, d)** in muscles injured in different ways. *P < 0.05, **P < 0.01. Rag, recombinase-activating gene.

There was no difference in the number of donor cells and donor muscle fibres between cryodamaged and irradiated+cryodamaged muscles, regardless of the muscle stem cell type grafted (Table 1, Figures 1 and 3).

### The number of nuclei and muscle fibres of donor origin is affected by the host mouse strain

To compare the transplantation efficiency in both host mouse strains, we transplanted the same CD133+ cells at the same time into Rag2-/γ chain-/C5- and *mdx* nude mice (experiment 4, Additional file 1: Table S1, Table 2). Within *mdx* nude hosts, there were similar numbers of nuclei and muscle fibres of donor origin as in the previous experiment (experiment 2, Additional file 1: Table S1; Table 1).

**Table 2:** **CD133+ donor cells have a higher transplantation efficiency in Rag2-/γ chain-/C5- than in** ***mdx*** **nude mice**

**Donor contribution**	**Injury model**	**Host mouse**		***P*** **value (Mann-Whitney test)**
		**Number of donor fibres (mean ± SEM (** ***n*** **))**		
		**Rag2-/γ chain-/C5-**	***mdx*** **nude**	
Human lamin A/C+ nuclei	Irradiation	98.83 ± 44.22 (6)	57.67 ± 39.76 (6)	0.3281
	Cryodamage	623.2 ± 117.3 (6)	184.8 ± 42.52 (6)	0.0087 **
	Irra+Cryo	569.3 ± 104.8 (6)	126.7 ± 34.66 (6)	0.0087**
S+L fibres	Irradiation	18.00 ± 6.583 (6)	19.17 ± 12.02 (6)	0.7440
	Cryodamage	191.3 ± 37.04 (6)	87.67 ± 13.10 (6)	0.0152*
	Irra+Cryo	146.7 ± 18.38 (6)	54.17 ± 19.53 (6)	0.0087**

*P < 0.05;**P < 0.01.

Within irradiated host muscles, there were no differences (*P* = 0.5045) in the number of human lamin A/C+ nuclei between Rag2-/γ chain-/C5- (98.83 ± 44.22) and *mdx* nude (57.67 ± 39.76) hosts. Also, there were no differences (*P* = 0.9339) in the number of S+L fibres between Rag2-/γ chain-/C5- (18.00 ± 6.583) and *mdx* nude (19.17 ± 12.02) mice.

When the host muscles were cryodamaged prior to grafting, there were significantly more human lamin A/C+ nuclei (*P* = 0.0056) in Rag2-/γ chain-/C5- mice (623.2 ± 117.3) than in *mdx* nude mice (184.8 ± 42.52) and significantly more S+L fibres (*P* = 0.0248) in Rag2-/γ chain-/C5- (191.3 ± 37.04) than in *mdx* nude (87.67 ± 13.10) mice.

**Figure 4:**
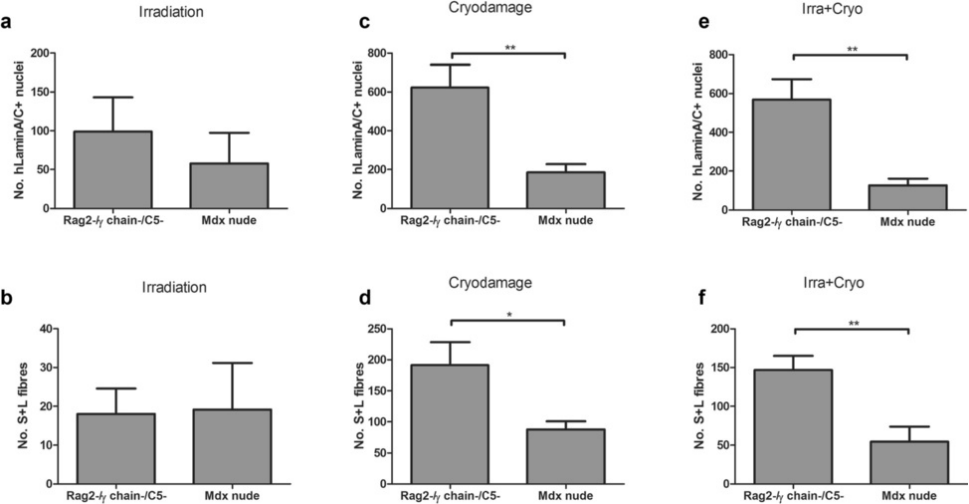
Donor cells have a significantly higher transplantation efficiency in degenerating and regenerating muscles in Rag2-/γ chain-/C5- than in *mdx* nude host mice. Quantification and comparison of the number of human lamin A/C+ nuclei and S+L fibres derived from donor CD133+ cells in irradiated **(a, b)**, cryodamaged **(c, d)** and irradiated+cryodamaged **(e, f)** muscles of Rag2-/γ chain-/C5- and *mdx* nude mice. *P < 0.05, **P < 0.01. Rag, recombinase-activating gene.

When the host muscles were irradiated+cryodamaged before cell transplantation, there were significantly more human nuclei (*P* = 0.0025) in Rag2-/γ chain-/C5- mice (569.3 ± 104.8) than in *mdx* nude mice (126.7 ± 34.66) and significantly more fibres of human origin (*P* = 0.0062) in Rag2-/γ chain-/C5- mice (146.7 ± 18.38) than in *mdx* nude (54.17 ± 19.53) mice (Figures 1 and 4, Table 2).

### Donor CD133+ cells give rise to more myogenic precursor (Pax7+) cells in Rag2-/γ chain-/C5- mice than in *mdx* nude host mice

To investigate the formation of myogenic cells (satellite cells and their progeny, myoblasts) derived from the transplanted donor cells, we compared the total number of Pax7+ myogenic cells and myogenic cells of host (Pax7+/human lamin A/C-) or donor (Pax7+/human lamin A/C+) origin, within grafted muscles in Rag2-/γ chain-/C5- and *mdx* nude mice (Table 3).

**Table 3:** **Pax7+ myogenic cells in muscles of Rag2-/γ chain-/C5- and** ***mdx*** **nude mice that had been grafted with CD133+ cells**

	**Injury models**	**Host mouse (mean ± SEM (** ***n*** **))**		***P*** **value (Mann-Whitney test)**
		**Rag2-/g chain-/C5-**	***mdx*** **nude**	
Total Pax7+ cells	Cryodamage	9.508 ± 1.933 (6)	8.071 ± 1.205 (6)	0.3095
	Irra+Cryo	3.320 ± 0.7494 (6)	3.464 ± 0.7909 (6)	0.9372
Host Pax7+ cells	Cryodamage	7.392 ± 1.381 (6)	87.67 ± 13.10 (6)	0.9372
	Irra+Cryo	0.7452 ± 0.217 (6)	3.019 ± 0.738 (6)	0.0411*
Donor Pax7+ cells	Cryodamage	2.116 ± 0.7793 (6)	0.5501 ± 0.3004 (6)	0.0931
	Irra+Cryo	2.574 ± 0.8427 (6)	0.4451 ± 0.2199 (6)	0.0152*
Percentage of donor Pax7+ cells	Cryodamage	20.67 ± 4.430 (6)	5.868 ± 2.840 (6)	0.026*
	Irra+Cryo	67.98 ± 10.45 (6)	11.46 ± 5.248 (6)	0.0064**
Donor-derived satellite cells	Cryodamage	1.400 ± 0.6211 (6)	0.2220 ± 0.1234 (6)	0.0303*
	Irra+Cryo	0.7798 ± 0.2294 (6)	0.1331 ± 0.0474 (6)	0.0173*

*P < 0.05; **P < 0.01.

Within irradiated host muscles, five out of six muscles in Rag2-/γ chain-/C5- mice contained donor-derived Pax7+ cells, and only two out of six muscles in *mdx* nude mice contained donor-derived Pax7+ cells. The *n* number in the latter group was too low for statistical analysis. Therefore, we only compared the formation of Pax7+ cells in cryodamaged or irradiated+cryodamaged muscles between the two mouse strains.

**Figure 5:**
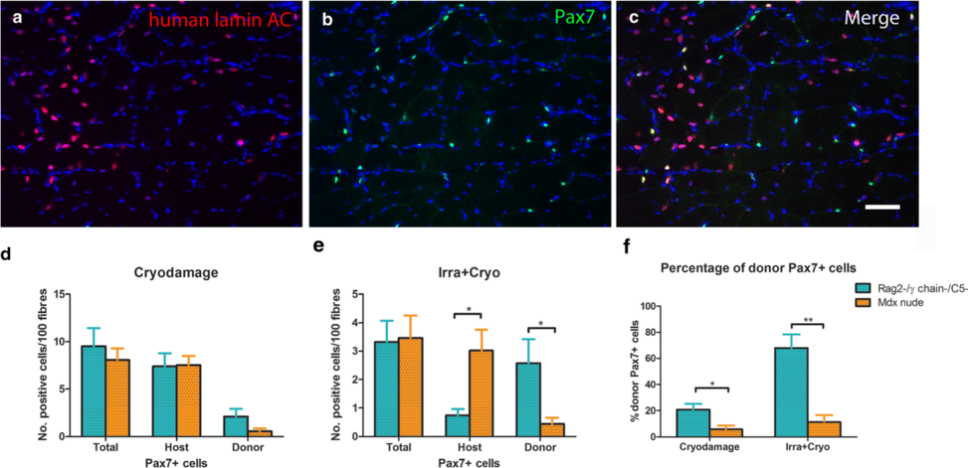
Myogenic cells of donor origin within regenerated host muscles. **(a-c)** Representative images of transverse cryosections of Rag2-/γ chain-/C5- host muscles that had been cryodamaged and transplanted with human CD133+ cells, stained with antibodies to human lamin A/C (**(a)**, red) and Pax7 (**(b)**, green). Nuclei were counterstained with DAPI (blue). **(c)** Merged image of human lamin A/C (red), Pax7 (green) and DAPI (blue). Scale bar = 25 μm. **(d)** The number of Pax7+ cells, Pax7+/human lamin A/C+ and Pax7+/human lamin A/C- cells per 100 muscle fibres in cryodamaged muscles of Rag2-/γ chain-/C5- and *mdx* nude mice. **(e)** the number of Pax7+ cells, Pax7+/human lamin A/C+ and Pax7+/human lamin A/C- cells per 100 muscle fibres in irradiated+cryodamaged muscles of Rag2-/γ chain-/C5- and *mdx* nude mice. **(f)** Percentage of donor-derived myogenic cells in each injury model. *P < 0.05, **P < 0.01. Rag, recombinase-activating gene.

When the host muscles were cryodamaged prior to cell transplantation, there was a similar total number (*P* = 0.5421) of myogenic cells/100 fibres in Rag2-/γ chain-/C5- (9.508 ± 1.933) and *mdx* nude (8.071 ± 1.205) mice. There were 7.392 ± 1.381 and 7.670 ± 13.10 myogenic cells of host origin/100 fibres in Rag2-/γ chain-/C5- and *mdx* nude mice, respectively, with no difference (*P* = 0.9409) between the two mouse strains. There were 2.116 ± 0.779 donor-derived myogenic cells/100 fibres in Rag2-/γ chain-/C5- mice and 0.550 ± 0.3 in *mdx* nude mice, representing 20.67% ± 4.430% and 5.868% ± 2.840% of the total myogenic cells in Rag2-/γ chain-/C5- and *mdx* nude mice, respectively. There were no statistically significant differences in the number (*P* = 0.0904) of donor-derived myogenic cells/100 fibres between the two mouse strains; however, there was a significantly higher percentage (*P* = 0.0184) of donor myogenic cells/100 fibres in Rag2-/γ chain-/C5- than *mdx* nude mice (Figure 5).

When the host muscles were irradiated+cryodamaged prior to cell transplantation, there was a similar total number (*P* = 0.9372) of myogenic cells/100 fibres in Rag2-/γ chain-/C5- (3.320 ± 0.7494) and *mdx* nude (3.464 ± 0.7909) mice. There were significantly fewer (*P* = 0.0144) myogenic cells of host origin/100 fibres in Rag2-/γ chain-/C5- (0.7452 ± 0.217) than *mdx* nude (3.019 ± 0.7382) mice, whereas there were significantly more (*P* = 0.0346) donor-derived myogenic cells/100 fibres in Rag2-/γ chain-/C5- (2.574 ± 0.8427) than *mdx* nude (0.4451 ± 0.2199) mice, representing 67.98% ± 10.45% and 11.46% ± 5.248%, respectively, of the total myogenic cells in Rag2-/γ chain-/C5- and *mdx* nude mice. There was a significantly higher percentage (*P* = 0.0007) of donor myoblasts in Rag2-/γ chain-/C5- than *mdx* nude mice (Figure 5).

### Donor CD133+ cells make a greater contribution to the stem cell pool in Rag2-/γ chain-/C5- than *mdx* nude host muscles

**Figure 6:**
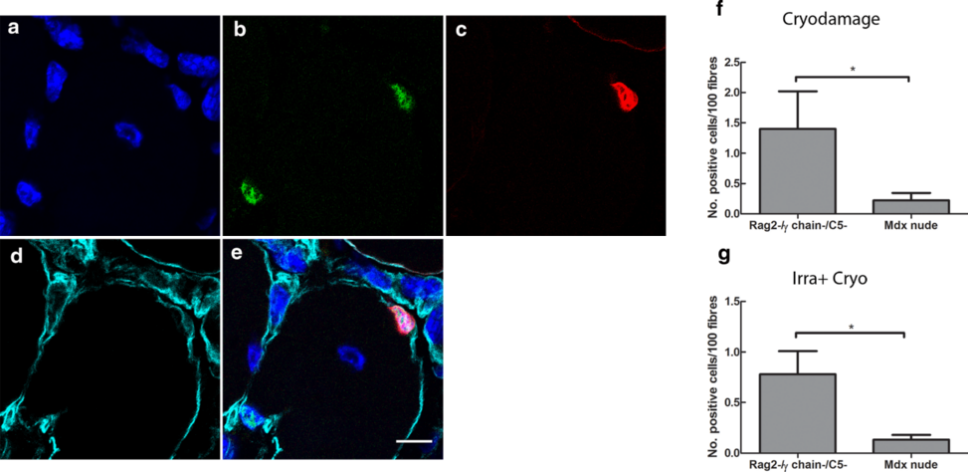
CD133+ cells contribute to more satellite cells in cryodamaged Rag2-/γ chain-/C5- than in *mdx* nude muscle. **(a-e)** Representative images showing donor-derived satellite cells that express Pax7 (green, **(b)**) and human lamin A/C (red, **(c)**), located underneath the basal lamina (cyan, **(d)**) of the muscle fibres. Nuclei were counterstained with DAPI (blue, **(a)**). (e) The merged image of **(a-d)**. Scale bar = 10 μm. **(f)** and **(g)**: quantification of the percentage of donor derived satellite cells in cryodamaged **(f)** or irradiated+cryodamaged (Irra+Cryo, **(g)**) muscles of Rag2-/γ chain-/C5- and *mdx* nude mice. *P < 0.05. Rag, recombinase-activating gene.

The above data compared the number of Pax7+ cells, which includes activated myogenic precursor cells and quiescent satellite cells. To evaluate the potential of donor cells to reconstitute the muscle stem cell pool within the different host muscle environments, we further quantified the percentage of grafted cells that are satellite cells. As pericytes do not give rise to Pax7+ cells within grafted muscles (Additional file 2: Figure S1), satellite cells of donor origin (Pax7+/human lamin A/C+ cells located underneath the basal lamina) in muscles that were transplanted with CD133+ cells were quantified and compared between Rag2-/γ chain-/C5- and *mdx* nude hosts. Again, we only compared the number of donor derived satellite cells in cryodamaged or irradiated+cryodamaged muscles (Figure 6).

In cryodamaged muscles, there were 1.400 ± 0.6211 donor-derived satellite cells/100 muscle fibres in Rag2-/γ chain-/C5- hosts, whereas in the *mdx* nude, there were only 0.2220 ± 0.1234 donor-derived satellite cells/100 fibres. There were significant differences (*P* = 0.0303) in the number of donor-derived satellite cells between the two mouse strains.

When muscles had been pre-injured by irradiation+cryodamage, the number of donor-derived satellite cells/100 fibres in Rag2-/γ chain-/C5- and *mdx* nude mice was 0.7798 ± 0.2294 and 0.1331 ± 0.0474, respectively, a significant difference between the two mouse strains (*P* = 0.0173).

## Discussion

Although different immunodeficient host mouse strains and different host muscle pre-injury regimes have been used by for human muscle stem cell transplantation (Rag2-/γ chain-/C5- hosts: irradiation or cryodamage; Rag1- and SCID hosts: irradiation followed by either BaCl_2_, or NaH_2_PO_4_, or cryodamage/crush, or cryodamage alone) and some appear to permit more efficient donor muscle formation than others [7,20], no comparison regarding the capability of donor stem cells to replenish the satellite cell pool in different animal models has been made. Here, we used two different types of human skeletal muscle stem cells - pericytes and CD133+ cells - as donor cells. We grafted these into two commonly used immunodeficient host mouse strains: *mdx* nude [31], whose muscles lack dystrophin and consequently undergo rounds of muscle degeneration and regeneration, and Rag2-/γ chain-/C5- mice [16], whose muscles have no pathology. In regenerated grafted muscles, we compared the number of nuclei, muscle fibres and satellite cells of donor origin.

### Effects of the injury models

How the host muscle is best modulated to promote efficient donor cell engraftment is not clear. This may depend on the pathological status of the host muscle or the type or species of origin of the donor cells. Irradiation kills most of the host satellite cells but spares the muscle fibres and preserves the satellite cell niche [14]. Cryodamage kills muscle fibres and most satellite cells but would also activate surviving host satellite cells. The combination of 18-Gy irradiation and cryodamage would destroy muscle fibres and kill most of the host satellite cells, thus ablating the satellite cell niche. But in irradiated muscles, radiation-resistant stem cells contribute to muscle regeneration following injury [14,37]. Each model therefore provides different environmental cues to donor cells.

We found that there were significantly more nuclei and muscle fibres of donor origin in host muscles that had been modulated by cryoinjury, or cryoinjury+irradiation, than by irradiation alone. This is host mouse strain independent, suggesting that in cryodamaged and irradiated+cryodamaged muscles, environmental changes are more permissive for human muscle stem cells than in irradiated muscles. In addition to degeneration and regeneration, massive infiltration of myeloid cells elicited by muscle injury is a factor known to control the fate of donor human myoblasts [18,38] and mouse mesoangioblasts [39,40]. Interaction between human pericytes or CD133+ cells and myeloid populations in a muscle environment are largely unknown, but our data suggest these muscle stem cells, as do myoblasts, respond to inflammatory stimuli.

Irradiation is known to promote the proliferation of donor mouse myoblasts [41], and this may be mediated by an alteration in the composition of the extracellular matrix [13]. However, in comparison with cryoinjury, irradiation of the host muscle 3 days before donor human stem cell transplantation does little or nothing to augment donor contribution to regenerated host muscle (Figures 1 and 2 and Tables 1 and 2). This is in contrast to our previous findings on mouse satellite cell transplantation into *mdx* nude hosts [14]. Also in contrast to donor mouse satellite cells [14], donor human skeletal muscle-derived stem cells function well within a cryoinjured host muscle environment. One possible reason for this is that donor cells from different species react differently to pro-inflammatory factors within the injured host muscle environment [42].

Our data clearly show that cryodamage prior to cell transplantation effectively elicits robust donor cell engraftment and that combining irradiation with cryodamage has no additive effect on the contribution of transplanted cells to muscle regeneration. However, the percentage of Pax7+ cells of donor origin was significantly higher in irradiated+cryodamaged than in cryodamaged Rag2-/γ chain-/C5- host mouse muscles (Figure 5f).

### Effects of the mouse strain

Different mouse strains present different environments for donor human muscle stem cells. Rag2-/γ chain-/C5- mice have a more profound immunodeficiency than the *mdx* nude mouse, while the latter is dystrophin deficient and its muscles exhibit pathological features, such as muscle degeneration and regeneration, accompanied by the infiltration of inflammatory cells.

The influence of the host mouse strain on transplantation efficiency was most clearly shown when the host muscle was challenged with profound damage, that is, cryodamage or irradiation+cryodamage. Under such conditions, there were significantly more nuclei, muscle fibres and satellite cells of donor origin in Rag2-/γ chain-/C5- than *mdx* nude mouse.

An interesting finding was that the ratio of the donor and host-derived Pax7+ cells within the grafted muscles was also different. Within transplanted cryoinjured muscles in both host stains, the majority of the muscle stem/precursor cell pool was host derived (Figure 5d). However, in irradiated+cryodamaged muscles, the Pax7+ cells were mainly of donor origin in Rag2-/γ chain-/C5- mice but were mainly of host origin in *mdx* nude mice (Figure 5e).

These differences in transplantation efficiency between the two strains may be due either to differences in their genetic background (both host mouse strains are on mixed, and different, genetic backgrounds), their immunological status or the pathological state of the host muscle. Further studies focusing on identifying the main factors responsible for augmenting donor muscle and satellite cell formation within dystrophic muscle are required to enable a long-term and effective stem cell therapy to DMD.

Although we and others [11] have shown that donor CD133+ cells derived from non-dystrophic donor muscle gave rise to Pax7+ cells following their engraftment, pericytes derived from DMD skeletal muscle did not. It remains to be seen whether CD133+ cells derived from DMD muscle can give rise to satellite cells. But satellite cells from *mdx* muscles give rise to functional satellite cells when transplanted into a permissive environment [43] - evidence that stem cells derived from a dystrophic muscle environment can be perfectly functional.

## Conclusions

The transplantation efficiency of human muscle stem cells is affected by both the local host mouse muscle environment and the host mouse strain. Cryoinjured muscles of Rag2-/γ chain-/C5- mice provide the most permissive environment for testing the contribution of intra-muscularly transplanted human skeletal muscle-derived stem cells to muscle regeneration. Although *mdx* nude host mouse muscles provide an environment in which there is significantly less donor human stem cell engraftment than do Rag2-/γ chain-/C5- mice, they nevertheless still permit donor-derived muscle regeneration. The advantage of such a dystrophin-deficient model is that the functionality of dystrophin restored by normal donor stem cells can be investigated.

## Additional files

Additional file 1: Table S1. Details of transplantation experiments. Details of the donor cells, host mice, muscle injury models and numbers of transplanted muscles in each experiment. Additional file 2: Figure S1. Human pericytes do not give rise to Pax7+ myogenic cells following their transplantation in vivo. Representative images showing the co-immunostaining of human lamin A/C, human spectrin (a, both red) and Pax7 (b, green) on transverse cryosections of Rag2-/γ chain-/C5- host muscles that had been cryodamaged and transplanted with human pericytes. There were no human lamin A/C+/Pax7+ nuclei present within the muscle. Nuclei were counterstained with DAPI (c, blue). Scale bar = 50 μm.

## Abbreviations

DAPI: 4′,6-diamidino-2-phenylindole
DMD: Duchenne muscular dystrophy
PFA: paraformaldehyde
Rag: recombinase-activating gene
S+L fibres: Human spectrin and human lamin A/C positive fibres
SCID: severe combined immune deficient
TA: tibialis anterior
