# Supplementary material for: The effect of the muscle environment on the regenerative capacity of human skeletal muscle stem cells
Source: Skelet Muscle. 2015 Apr 28;5:11. doi: 10.1186/s13395-015-0036-8 (PMC4422426; doi:10.1186/s13395-015-0036-8)
Supplement: Additional file 1: Table S1. — Details of transplantation experiments. Details of the donor cells, host mice, muscle injury models and numbers of transplanted muscles in each experiment. [file 13395_2015_36_MOESM1_ESM.docx]

**Supplementary Table S1. Details of transplantation experiments.**

|  | | | | | |  | |
| --- | --- | --- | --- | --- | --- | --- | --- |
| Experiment number | Donor cell type | Mpds | Host mouse | Injury model (n number) | | | |
|  |  |  |  | Irradiation | Cryodamage | | Irra+Cryo |
| 1 | Pericytes | 18.9 | *Mdx* nude | 6 | 6 | | 5 |
| 2 | CD133+ cells | 6.8 | *Mdx* nude | 6 | 6 | | 5 |
| 3 | Pericytes | 13.58 | Rag2-/γ chain-/C5- | 11 | 8 | | 11 |
| 4 | CD133+ cells | 7.91 | Rag2-/γ chain-/C5- | 6 | 6 | | 6 |
|  |  |  | *Mdx* nude | 6 | 6 | | 6 |
