# Supplementary figures and images for: The effect of the muscle environment on the regenerative capacity of human skeletal muscle stem cells
Source: Skelet Muscle. 2015 Apr 28;5:11. doi: 10.1186/s13395-015-0036-8 (PMC4422426; doi:10.1186/s13395-015-0036-8)

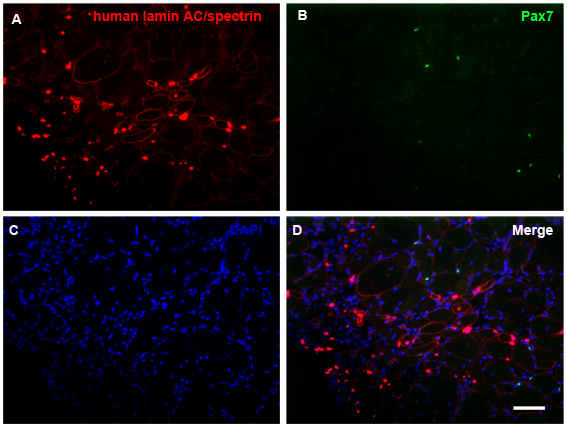

Supplement: Additional file 2: Figure S1. — Human pericytes do not give rise to Pax7+ myogenic cells following their transplantation in vivo. Representative images showing the co-immunostaining of human lamin A/C, human spectrin (a, both red) and Pax7 (b, green) on transverse cryosections of Rag2-/γ chain-/C5- host muscles that had been cryodamaged and transplanted with human pericytes. There were no human lamin A/C+/Pax7+ nuclei present within the muscle. Nuclei were counterstained with DAPI (c, blue). Scale bar = 50 μm. [file 13395_2015_36_MOESM2_ESM.tif]
